# RETRACTED: Sudakov et al. Cross-Generational Impact of Epigenetic Male Influence on Physical Activity in Rat. *Biology* 2022, *11*, 1606

**DOI:** 10.3390/biology14081085

**Published:** 2025-08-20

**Authors:** Sergey K. Sudakov, Natalia G. Bogdanova, Galina A. Nazarova

**Affiliations:** P.K. Anokhin Research Institute of Normal Physiology, 125315 Moscow, Russia

The *Biology* Editorial Office retracts the paper entitled “Cross-Generational Impact of Epigenetic Male Influence on Physical Activity in Rat” [1], cited above.

Following publication, concerns were brought to the attention of the Editorial Office regarding major scientific errors and the overall validity of the findings presented in this article [1].

Adhering to our complaints procedure, an investigation was conducted by the Editorial Office and Editorial Board that confirmed the presence of scientific errors within this study. Furthermore, the Editorial Board determined that the authors had failed to appropriately notify the journal that portions of the data presented had previously been retracted [2]. As a result, the Editorial Board has lost confidence in the reliability of the findings and has decided to retract this publication [1], as per MDPI’s retraction policy (https://www.mdpi.com/ethics#_bookmark30).

This retraction was approved by the Editors-in-Chief of the journal *Biology*.

The authors did not agree with this retraction.
